# Reproduction of EEG power spectrum over frontal region during the propofol-induced general anesthesia

**DOI:** 10.1186/1471-2202-15-S1-P211

**Published:** 2014-07-21

**Authors:** Meysam Hashemi, Axel Hutt, Jamie Sleigh, Peter beim Graben

**Affiliations:** 1 INRIA CR Nancy - Grand Est, Villers-les-Nancy, France; 2 Department of Anaesthetics, Waikato Hospital, Hamilton, New Zealand; 3 Department of German Language and Linguistic, Hamboldt-Universitat zu Berlin, Germany

## 

The present work aims to reproduce certain changes observed experimentally in the EEG power spectrum over the frontal head region during general anesthesia induced by propofol. These observations include increased delta (0-4 Hz) and alpha (8-12 Hz) activities [1]. We extend a previous cortical model [2] and study a neuronal population model of a single thalamo-cortical module consisting of three different populations of neurons, namely cortical excitatory neurons, thalamocortical relay neurons and inhibitory thalamic reticular neurons (Fig. 1). Each module obeys a neural mass model. The cortical inhibitory population is neglected in our model to reveal the effect of propofol action in the thalamus. Our model describes well the characteristic spectral changes observed experimentally within the delta- and alpha- frequency bands in frontal and occipital electrodes with increasing concentration of propofol.

**Figure 1:**
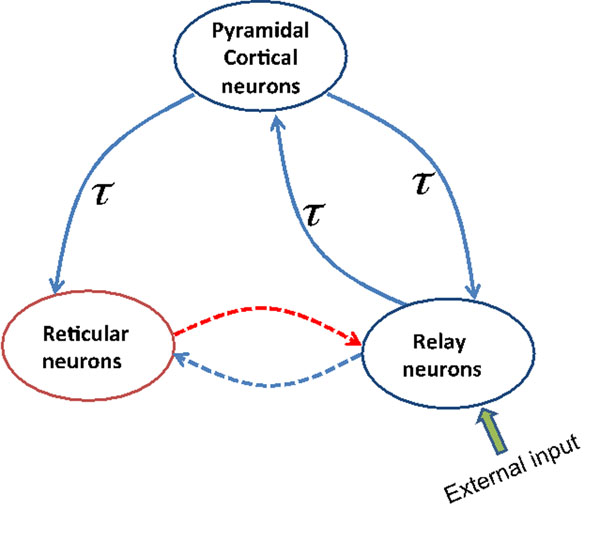
Schematic of a thalamocortical module. The blue and red lines indicate excitatory and inhibitory connections, respectively. The solid lines represent connections associated with the same delay and the dotted lines denote connections without delay.

This shows that neglecting inhibitory action in the cortex but considering thalamic GABAergic action suffices to reproduce the data. From a modeling point of view, our reduced mathematical model is low dimensional and remains analytically treatable while still being adequate to reproduce observed changes in EEG rhythms. Moreover, it is shown that the propofol concentration acts as a control parameter of the system and that propofol-induced changes in the stationary states of the model system lead to changes in the corresponding nonlinear gain function that result in EEG power modulation: increases of power over the frontal region can be caused by an increase in the gain function of thalamocortical network. The results suggest that intra-thalamic inhibition from reticular neurons to relay cells plays an important role in the generation of the characteristic EEG patterns seen during general anesthesia.

## References

[B1] Cimenser A Tracking brain states under general anesthesia by using global coherence analysis PNAS 2011 15 8832 8837 21555565 10.1073/pnas.1017041108 PMC3102391

[B2] Hutt A The anaesthetic propofol shifts the frequency of maximum spectral power in EEG during general anaesthesia: analytical insights from a linear model, Front. Comput. Neurosci 2013 15 2 23386826 10.3389/fncom.2013.00002 PMC3564209

